# Association between metabolic score for insulin resistance and cardiovascular disease mortality in patients with rheumatoid arthritis: evidence from the NHANES 1999-2018

**DOI:** 10.3389/fendo.2024.1444800

**Published:** 2024-09-13

**Authors:** Yan Zhou, Jie Gao

**Affiliations:** ^1^ Clinical Laboratory, Shanxi Bethune Hospital, Shanxi Academy of Medical Sciences, Tongji Shanxi Hospital, Third Hospital of Shanxi Medical University, Taiyuan, China; ^2^ Tongji Hospital, Tongji Medical College, Huazhong University of Science and Technology, Wuhan, China

**Keywords:** cardiovascular disease, metabolic score for insulin resistance, mortality, prognosis, rheumatoid arthritis

## Abstract

**Aim:**

To explore the association between Metabolic Score for Insulin Resistance (METS-IR) and the risk of cardiovascular disease (CVD) death in patients with rheumatoid arthritis (RA).

**Methods:**

This retrospective cohort study extracted data on 1,218 RA patients from the National Health and Nutrition Examination Survey. The weighted univariate and multivariate Cox regression model was established to explore the association between METS-IR and CVD mortality. Subgroup analysis was performed in terms of age, gender, body mass index, diabetes, and CVD. Hazard ratios (HRs) and 95% confidence levels (CIs) were presented.

**Results:**

Increased METS-IR was associated with a significantly higher risk of CVD mortality (HR=4.59, 95%CI: 1.98-10.67), and METS-IR>2.48 was associated with higher odds of CVD mortality compared with METS-IR ≤ 2.25 (HR=3.57, 95%CI: 2.04-6.24). METS-IR was positively associated with the risk of CVD mortality (HR=3.83, 95%CI: 1.62-9.08), and METS-IR>2.48 was associated with a significantly higher risk of CVD mortality in contrast to METS-IR ≤ 2.25 (HR=3.38, 95%CI: 1.87-6.09).

**Conclusion:**

Increased METS-IR was associated with a significantly higher risk of CVD mortality in RA patients. Clinicians could consider incorporating the METS-IR score into routine assessment of the prognosis of RA patients.

## Introduction

Rheumatoid arthritis (RA) is an autoimmune joint disease related to synovial tissue proliferation, pannus formation, cartilage destruction, and systemic complications (1, 2), with a global prevalence of 460 per 100,000 people from 1980 to 2019 and a moderately increased mortality risk over the last two decades (3, 4). Individuals with RA have 1.5-2 times the rate of cardiovascular events than the general population, and cardiovascular events are the primary cause of death in people with RA (5). As evidence shows, RA patients have a 60% increased risk of cardiovascular disease (CVD) mortality compared with the general population (6–9).

Insulin resistance is an important factor affecting the occurrence and prognosis of CVD (10–12). Studies reported that insulin resistance may play a critical role in the association between RA and cardiovascular disease (13, 14). Insulin resistance significantly increases the risk of atherosclerosis and CVD in patients with RA (15–17).

The current evaluation methods for insulin resistance (IR) are diverse. The euglycemic-hyperinsulinemic clamp (EHC) is considered the gold standard, but its invasive and costly limitations make it unsuitable for large-scale clinical and epidemiological studies (18). Thus, researchers have developed several indices to assess insulin resistance using simple formulas, such as the homeostasis model assessment for IR (HOMA-IR) (19), triglyceride (TG) glucose index (20), and TG to high-density lipoprotein cholesterol ratio (HDL-C) (TG/HDL-C) (21). However, the aforementioned indexes fail to account for the impact of nutritional factors, such as body mass index (BMI), on insulin resistance. Consequently, they possess certain limitations when constructing clinical disease prediction models. Recently, a tool based on fasting blood glucose (FBG), TG, HDL-C, and BMI, namely Metabolic Score for Insulin Resistance (METS-IR), has been demonstrated to be effective in evaluating insulin resistance (22). METS-IR was illustrated to be correlated with adipokine disorder and inflammatory activity in patients with osteoarthritis (23), and the higher METS-IR in patients with diabetes was associated with the increased risk of CVD mortality (24). Therefore, we suspected that METS-IR might be associated with the CVD mortality in RA patients.

This study aimed to explore the association between METS-IR and the risk of CVD death in patients with RA, using the data of the National Health and Nutrition Examination Survey (NHANES). This association was further investigated in different age, gender, body mass index (BMI), diabetes, and CVD subpopulations.

## Methods

### Study population

This retrospective cohort study extracted data on patients with RA from the NHANES 1999-2018. The NHANES is a program of studies conducted to evaluate the health and nutritional status of the nationally representative population in the United States, which combines interviews and physical examinations to provide information on demographics, diets, physical examinations, laboratory tests, and questionnaire surveys (25). The Ethics Review Board of the National Center for Health Statistics (NCHS) Research approves the NHANES survey. This study was exempt from the approval of the institutional review board due to de-identified and retrospective nature of the data used. Patient records were obtained from NHANES if they 1) were ≥18 years old; 2) had RA. RA in this study was self-reported by physicians. The positive answer for the question “Doctor ever said you had arthritis?” and the “rheumatoid arthritis” answer for the question “Which type of arthritis was it” indicated the presence of RA. The above questionnaire was completed by professionally trained investigators under strict quality control (26, 27); 3) had measurement of FBG, TG and HDL-C, 4) had measurement of height and weight; and 5) had information on survival status and death causes. Patients with missing important co-variables were excluded from this study. Qualified patients were followed up until December 31, 2019.

### Main and outcome variables

METS-IR was calculated as ln [(2 × FPG (mg/dL) + TG (mg/dL)) × BMI (kg/m^2^)]/ln [HDL-C (mg/dL)] (22). It was treated as both a continuous variable and a categorical variable. When METS-IR acted as a continuous variable, it was classified into three groups (≤2.25, 2.25-2.48, and >2.48).

The outcome in this study was CVD mortality. The follow-up was ended in December 2019. The mean follow-up time was 125.36 ± 3.99 months.

### Other variables

Age (<65, ≥65 years), gender (male, female), race (non-Hispanic White, non-Hispanic Black, others), education [below high school, high school, college and above], marital status (married, never married, others), poverty income ratio (PIR; <1.0, ≥1.0), smoking (no, yes), drinking (no, yes), total energy (kcal), physical activity, duration of arthritis (years), osteoporosis (no, yes, unknown), fracture (no, yes, unknown), diabetes (no, yes), hypertension (no, yes), dyslipidemia (no, yes), CVD, chronic kidney disease (CKD; no, yes), dyslipidemia (no, yes), BMI (kg/m^2^), white blood cell (WBC, 1000 cells/μL), uric acid (mg/dL), antirheumatics (no, yes), nonsteroidal anti-inflammatory agents (no, yes), and glucocorticoid (no, yes) were also analyzed.

Physical activity was exhibited as energy consumption (MET × min), which was calculated by multiplying recommended metabolic equivalent (MET) by exercise time corresponding to the activity (min). Then physical activity was divided into <450 MET × min/week, ≥450 MET × min/week and unknown.

### Statistical analysis

Quantitative data were reported as mean (standard error) [Mean (SE)], the independent samples t-test was used for comparison between two groups, and analysis of variance was applied for comparison among multiple groups; Enumeration data were shown as the number and percentages of cases [n (%)], and the Chi-square test was used for comparison among groups and the Mann-Whitney U rank sum test was employed for ranked data. The missing values were shown in **Supplementary Table 1**. The missing values concerning physical activity, osteoporosis and fracture were high, and patients without these variables data were classified as unknown group. The other variables dealt with multiple imputation method based on random forest using the miceforest package in python, and differences between pre- and post-imputation data were assessed (**Supplementary Table 2**). The weighted univariate Cox regression model was used to explore the factors which were significantly associated with CVD mortality in RA patients, which would be utilized as confounding variables. Then the weighted univariate and multivariate Cox regression model was established to explore the association between METS-IR and the risk of CVD mortality. Model I adjusted for age, education, marital status, physical activity, duration of arthritis, WBC, and uric acid. Model II adjusted for age, education, marital status, physical activity, duration of arthritis, WBC, uric acid, diabetes, hypertension, dyslipidemia, CVD, and CKD. Further, subgroup analysis was performed in terms of age, gender, BMI, diabetes, and CVD. Hazard ratios (HRs) and 95% confidence levels (CIs) were presented. SAS 9.4 (SAS Institute Inc., Cary, NC, USA) was applied for model statistical analysis. All statistical tests were two-sided, with *P* < 0.05 indicating statistically significant differences.

## Results

### Characteristics of the study population

**Figure 1:**
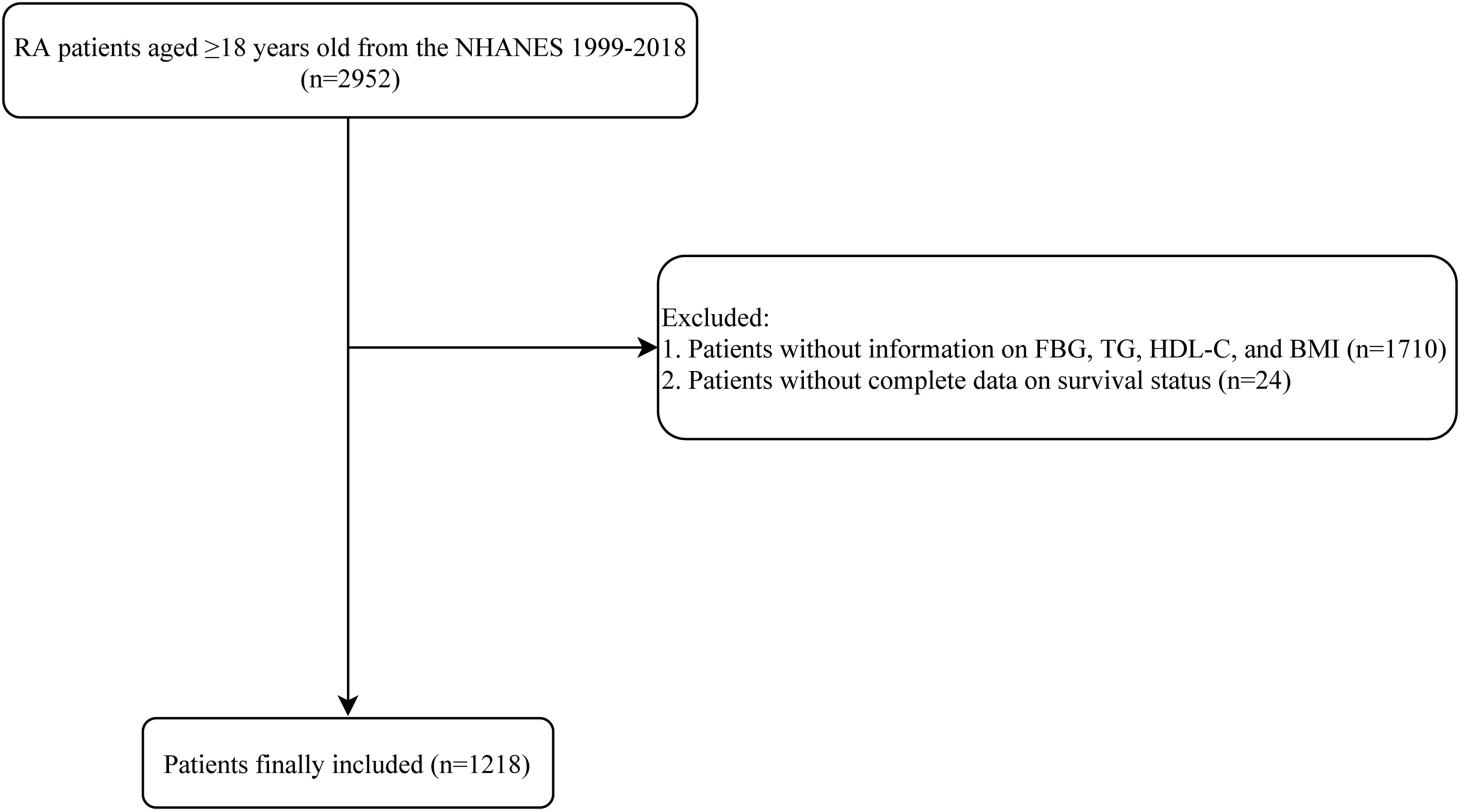
Process of study population selection. RA, rheumatoid arthritis; NHANES, National Health and Nutrition Examination Survey; HDL-C, high-density lipoprotein cholesterol; BMI, body mass index.

A total of 2952 patients with RA who aged ≥18 years old were identified from the NHANES 1999-2018. After ruling out patients without information on FBG, TG, HDL-C, and BMI (n=1710), and without complete data on survival status (n=24), 1218 patients were included in the end. The process of study population selection is illustrated in **Figure 1**.

At the end of follow-up. there were 129 patients died of CVD. The average age were 57.15 years. The CVD mortality group had significantly higher METS-IR, older age, and lower education level than the non-CVD mortality group (all *P*<0.05). There were significant differences in marital status, total energy, physical activity, duration of arthritis, osteoporosis, fracture, diabetes, hypertension, dyslipidemia, CVD, CKD, and uric acid between the CVD mortality and non-CVD mortality groups (all *P*<0.05). Characteristics of the included patients with RA are demonstrated in **Table 1**.

**Table 1:** Characteristics of the included patients with RA.

Variables	Total (n=1218)	Non-CVD mortality group (n=1089)	CVD mortality group (n=129)	Statistics	*P*
METS-IR, Mean (SE)	2.38 (0.01)	2.37 (0.01)	2.45 (0.03)	t=-3.08	0.002
METS-IR, n (%)				χ^2^ = 9.20	0.010
≤2.25	410 (32.93)	379 (34.00)	31 (20.90)		
2.25-2.48	418 (33.72)	371 (33.95)	47 (31.17)		
>2.48	390 (33.35)	339 (32.05)	51 (47.94)		
Age, years, Mean (SE)	57.15 (0.60)	56.28 (0.60)	66.99 (2.02)	t=-5.13	<0.001
Age, n (%)				χ^2^ = 22.72	<0.001
<65	699 (67.48)	665 (70.12)	34 (37.82)		
≥65	519 (32.52)	424 (29.88)	95 (62.18)		
Gender, n (%)				χ^2^ = 0.20	0.658
Male	522 (43.23)	467 (43.02)	55 (45.65)		
Female	696 (56.77)	622 (56.98)	74 (54.35)		
Race, n (%)				χ^2^ = 3.61	0.164
Non-Hispanic White	516 (66.06)	453 (65.36)	63 (73.96)		
Non-Hispanic Black	345 (17.07)	318 (17.36)	27 (13.79)		
Others	357 (16.87)	318 (17.28)	39 (12.24)		
Education, n (%)				χ^2^ = 7.86	0.020
Below high school	457 (27.22)	391 (26.19)	66 (38.87)		
High school	287 (27.17)	253 (26.74)	34 (32.08)		
College and above	474 (45.60)	445 (47.07)	29 (29.05)		
Marital status, n (%)				χ^2^ = 10.61	0.005
Married	642 (60.56)	579 (61.76)	63 (47.05)		
Never married	89 (6.67)	82 (6.86)	7 (4.61)		
Others	487 (32.77)	428 (31.39)	59 (48.34)		
PIR, n (%)				χ^2^ = 0.84	0.359
<1.0	305 (19.44)	264 (19.11)	41 (23.11)		
≥1.0	913 (80.56)	825 (80.89)	88 (76.89)		
Smoking, n (%)				χ^2^ = 0.31	0.577
No	529 (38.01)	468 (37.73)	61 (41.13)		
Yes	689 (61.99)	621 (62.27)	68 (58.87)		
Drinking, n (%)				χ^2^ = 0.70	0.404
No	455 (33.78)	398 (33.37)	57 (38.44)		
Yes	763 (66.22)	691 (66.63)	72 (61.56)		
Total energy, kcal, Mean (SE)	2002.83 (40.57)	2021.53 (41.80)	1792.48 (107.98)	t=2.07	0.041
Physical activity, n (%)				χ^2^ = 12.94	0.002
<450 MET × minutes/week	131 (12.10)	119 (11.62)	12 (17.51)		
≥450 MET × minutes/week	578 (51.53)	544 (53.48)	34 (29.56)		
Unknown	509 (36.37)	426 (34.89)	83 (52.94)		
Duration of arthritis, years, Mean (SE)	14.10 (0.50)	13.74 (0.52)	18.18 (1.79)	t=-2.35	0.020
Osteoporosis, n (%)				χ^2^ = 13.36	0.001
No	825 (64.74)	726 (63.63)	99 (77.24)		
Yes	140 (11.28)	118 (10.79)	22 (16.83)		
Unknown	253 (23.97)	245 (25.58)	8 (5.93)		
Fracture, n (%)				χ^2^ = 12.32	0.002
No	810 (61.36)	711 (60.25)	99 (73.86)		
Yes	156 (14.68)	134 (14.19)	22 (20.21)		
Unknown	252 (23.96)	244 (25.56)	8 (5.93)		
Diabetes, n (%)				χ^2^ = 6.50	0.011
No	820 (74.87)	747 (76.10)	73 (61.05)		
Yes	398 (25.13)	342 (23.90)	56 (38.95)		
Hypertension, n (%)				χ^2^ = 10.66	0.001
No	369 (36.83)	348 (38.52)	21 (17.79)		
Yes	849 (63.17)	741 (61.48)	108 (82.21)		
Dyslipidemia, n (%)				χ^2^ = 8.19	0.004
No	212 (18.01)	196 (18.81)	16 (9.05)		
Yes	1006 (81.99)	893 (81.19)	113 (90.95)		
CVD, n (%)				χ^2^ = 14.11	<0.001
No	697 (61.18)	648 (63.09)	49 (39.66)		
Yes	521 (38.82)	441 (36.91)	80 (60.34)		
CKD, n (%)				χ^2^ = 17.69	<0.001
No	935 (82.45)	855 (84.06)	80 (64.23)		
Yes	283 (17.55)	234 (15.94)	49 (35.77)		
BMI, kg/m^2^, Mean (SE)	30.60 (0.27)	30.53 (0.28)	31.40 (0.75)	t=-1.09	0.279
WBC, 1000 cells/uL, Mean (SE)	7.01 (0.09)	6.97 (0.09)	7.41 (0.23)	t=-1.74	0.083
Uric acid, mg/dL, Mean (SE)	5.74 (0.06)	5.70 (0.06)	6.22 (0.24)	t=-2.07	0.040
Antirheumatics, n (%)				χ^2^ = 3.77	0.052
No	1114 (89.00)	991 (88.51)	123 (94.56)		
Yes	104 (11.00)	98 (11.49)	6 (5.44)		
Nonsteroidal anti-inflammatory agents, n (%)				χ^2^ = 0.07	0.798
No	1052 (85.38)	941 (85.47)	111 (84.37)		
Yes	166 (14.62)	148 (14.53)	18 (15.63)		
Glucocorticoid, n (%)				χ^2^ = 0.03	0.864
No	1136 (91.72)	1018 (91.67)	118 (92.19)		
Yes	82 (8.28)	71 (8.33)	11 (7.81)		
Follow-up time, months, Mean (SE)	125.36 (3.99)	126.96 (4.18)	107.43 (9.74)	t=1.86	0.065

RA, rheumatoid arthritis; CVD, cardiovascular disease; METS-IR, Metabolic Score for Insulin Resistance; PIR, poverty income ratio; CKD, chronic kidney disease; BMI, body mass index; WBC, white blood cell; SE, standard error.

### Association between METS-IR and CVD mortality in RA

The confounding factors associated with CVD mortality in RA were screening via weighted univariate Cox regression model, which indicated that age, education, marital status, physical activity, duration of arthritis, WBC, uric acid, diabetes, hypertension, dyslipidemia, CVD, and CKD were confounding factors (**Supplementary Table 3**). After controlling for age, education, marital status, physical activity, duration of arthritis, WBC, and uric acid, increased METS-IR was associated with higher risk of CVD mortality (HR=4.59, 95%CI: 1.98-10.67, *P*<0.001), and METS-IR>2.48 was associated with a significantly greater risk of CVD mortality compared with METS-IR ≤ 2.25 (HR=3.57, 95%CI: 2.04-6.24, *P*<0.001). After adjusting for age, education, marital status, physical activity, duration of arthritis, WBC, uric acid, diabetes, hypertension, dyslipidemia, CVD, and CKD, METS-IR was associated with elevated risk of CVD mortality (HR=3.83, 95%CI: 1.62-9.08, *P*=0.003), and METS-IR>2.48 was associated with higher risk of CVD mortality in contrast to METS-IR ≤ 2.25 (HR=3.38, 95%CI: 1.87-6.09, *P*<0.001) (**Table 2**).

**Table 2:** Association between METS-IR and CVD mortality in RA.

Variables	Crude Model		Model I		Model II	
	HR (95%CI)	*P*	HR (95%CI)	*P*	HR (95%CI)	*P*
METS-IR	2.99 (1.51-5.95)	0.002	4.59 (1.98-10.67)	<0.001	3.83 (1.62-9.08)	0.003
METS-IR						
≤2.25	Ref		Ref		Ref	
2.25-2.48	1.42 (0.72-2.78)	0.306	1.67 (0.87-3.18)	0.121	1.68 (0.85-3.33)	0.138
>2.48	2.23 (1.29-3.87)	0.005	3.57 (2.04-6.24)	<0.001	3.38 (1.87-6.09)	<0.001

Crude Model: a univariate model.

Model I: adjusted for age, education, marital status, physical activity, duration of arthritis, WBC, and uric acid.

Model II: adjusted for age, education, marital status, physical activity, duration of arthritis, WBC, uric acid, diabetes, hypertension, dyslipidemia, CVD, and CKD.

RA, rheumatoid arthritis; CVD, cardiovascular disease; METS-IR, Metabolic Score for Insulin Resistance; CKD, chronic kidney disease; WBC, white blood cell; HR, hazard ratio; CI, confidence level.

### Association between METS-IR and CVD mortality in RA subpopulations

#### Age

In patients aged<65 years, higher METS-IR was associated with higher risk of CVD mortality (HR=2.63, 95%CI: 1.10-6.32, *P*=0.031); compared with METS-IR ≤ 2.25, METS-IR>2.48 was associated with higher risk of CVD mortality (HR=3.29, 95%CI: 1.69-6.39, *P*<0.001). For patients aged ≥65 years, METS-IR was associated with increased risk of CVD mortality (HR=5.48, 95%CI: 1.71-17.57, *P*=0.005), and METS-IR of 2.25-2.48 (HR=2.30, 95%CI: 1.03-5.16, *P*=0.043) and METS-IR>2.48 (HR=3.68, 95%CI: 1.62-8.40, *P*=0.002) were associated with higher risk of CVD mortality than METS-IR ≤ 2.25 (**Table 3**).

**Table 3:** Association between METS-IR and CVD mortality in RA subpopulations.

Subgroup	Model		Model	
	HR (95%CI)	*P*	HR (95%CI)	*P*
Subgroup I: Age	Age<65 years (n=699)		Age≥65 years (n=519)	
METS-IR	2.63 (1.10-6.32)	0.031	5.48 (1.71-17.57)	0.005
METS-IR				
≤2.25	Ref		Ref	
2.25-2.48	0.86 (0.24-3.06)	0.816	2.30 (1.03-5.16)	0.043
>2.48	3.29 (1.69-6.39)	<0.001	3.68 (1.62-8.40)	0.002
Subgroup II: Gender	Male (n=522)		Female (n=696)	
METS-IR	2.56 (0.50-13.12)	0.255	4.21 (1.75-10.13)	0.002
METS-IR				
≤2.25	Ref		Ref	
2.25-2.48	1.07 (0.47-2.45)	0.868	2.02 (0.86-4.71)	0.104
>2.48	2.03 (1.02-4.05)	0.044	4.01 (1.73-9.31)	0.001
Subgroup III: BMI	BMI<30 kg/m^2^ (n=661)		BMI≥30 kg/m^2^ (n=557)	
METS-IR	9.44 (1.99-44.77)	0.005	2.32 (0.81-6.64)	0.115
METS-IR				
≤2.25	Ref		Ref	
2.25-2.48	2.31 (1.13-4.73)	0.023	0.87 (0.28-2.65)	0.801
>2.48	4.03 (1.69-9.62)	0.002	2.29 (0.76-6.86)	0.138
Subgroup IV: Diabetes	No (n=820)		Yes (n=398)	
METS-IR	10.31 (3.16-33.63)	<0.001	1.74 (0.64-4.73)	0.269
METS-IR				
≤2.25	Ref		Ref	
2.25-2.48	1.23 (0.54-2.83)	0.619	2.16 (0.64-7.26)	0.210
>2.48	4.38 (2.44-7.86)	<0.001	2.62 (1.10-6.28)	0.031
Subgroup V: CVD	No (n=697)		Yes (n=521)	
METS-IR	5.61 (0.95-33.15)	0.057	3.30 (1.50-7.28)	0.004
METS-IR				
≤2.25	Ref		Ref	
2.25-2.48	1.32 (0.59-2.93)	0.498	1.98 (0.88-4.45)	0.098
>2.48	2.82 (1.36-5.85)	0.006	3.54 (1.81-6.93)	<0.001

Subgroup I/II/III: if not stratified, adjusted for age, education, marital status, physical activity, duration of arthritis, WBC, uric acid, diabetes, hypertension, dyslipidemia, CVD, and CKD.

Subgroup IV: if not stratified, adjusted for age, education, marital status, physical activity, duration of arthritis, WBC, uric acid, hypertension, dyslipidemia, CVD, and CKD.

Subgroup V: if not stratified, adjusted for age, education, marital status, physical activity, duration of arthritis, WBC, uric acid, diabetes, hypertension, dyslipidemia, and CKD.

RA, rheumatoid arthritis; CVD, cardiovascular disease; METS-IR, Metabolic Score for Insulin Resistance; CKD, chronic kidney disease; WBC, white blood cell; HR, hazard ratio; CI, confidence level.

#### Gender

For male patients, compared with METS-IR ≤ 2.25, METS-IR>2.48 was associated with higher risk of CVD mortality (HR=2.03, 95%CI: 1.02-4.05, *P*=0.044). Regarding female patients, METS-IR was related to elevated risk of CVD mortality (HR=4.21, 95%CI: 1.75-10.13, *P*=0.002), and METS-IR>2.48 were associated with higher risk of CVD mortality than METS-IR ≤ 2.25 (HR=4.01, 95%CI: 1.73-9.31, *P*=0.001) (**Table 3**).

#### BMI

Among patients with BMI<30 kg/m^2^, METS-IR was correlated with increased risk of CVD mortality (HR=9.44, 95%CI: 1.99-44.77, *P*=0.005), and METS-IR of 2.25-2.48 (HR=2.31, 95%CI: 1.13-4.73, *P*=0.023) and METS-IR>2.48 (HR=4.03, 95%CI: 1.69-9.62, *P*=0.002) were associated with higher risk of CVD mortality than METS-IR ≤ 2.25. No significant association between METS-IR and CVD mortality was identified in patients with BMI≥30kg/m^2^ (all *P*>0.05) (**Table 3**).

#### Diabetes

In patients with diabetes, elevated METS-IR was associated with higher risk of CVD mortality (HR=10.31, 95%CI: 3.16-33.63, *P*<0.001), and METS-IR>2.48 was associated with increased risk of CVD mortality compared with METS-IR ≤ 2.25 (HR=4.38, 95%CI: 2.44-7.86, *P*<0.001). For patients without diabetes, METS-IR>2.48 was associated with higher risk of CVD mortality than METS-IR ≤ 2.25 (HR=2.62, 95%CI: 1.10-6.28, *P*=0.031) (**Table 3**).

#### CVD

Concerning patients with CVD, METS-IR>2.48 was associated with increased risk of CVD mortality than METS-IR ≤ 2.25 (HR=2.82, 95%CI: 1.36-5.85, *P*=0.006). For patients without CVD, METS-IR was related to elevated risk of CVD mortality (HR=3.30, 95%CI: 1.50-7.28, *P*=0.004), and METS-IR>2.48 were associated with higher risk of CVD mortality than METS-IR ≤ 2.25 (HR=3.54, 95%CI: 1.81-6.93, *P*<0.001) (**Table 3**).

## Discussion

In this study, we explored the relationship between METS-IR and the risk of CVD mortality in patients with RA. It was demonstrated that increased METS-IR was associated with elevated risk of CVD mortality, and METS-IR>2.48 was associated with increased risk of CVD mortality compared with METS-IR ≤ 2.25. These findings offered the understanding of METS-IR and CVD mortality relationship, and could be considered in the clinical management of prognosis in RA patients.

In the recent studies, METS-IR was reported to be associated with elevated risk of CVD/stroke/cardiac issue in the middle-aged and elderly population (28). Wang et al. (24) illustrated that METS-IR was non-linearly associated with all-cause and CVD-related death in patients with diabetes. The correlation between METS-IR and adipokine disorder and inflammatory activity in females with knee osteoarthritis (23). METS-IR was also found to be significantly associated with all-cause and CVD mortality in the U.S. population compared to the other three alternative insulin resistance indexes (TyG index, TG/HDL-C, and HOMA-IR) (29). A cohort study reported that METS-IR was associated with elevated risk of stroke and ischemic stroke in patients with hypertension (30). Another large, prospective cohort study demonstrated that the METS-IR was independently associated with a higher risk of all-cause and CVD mortality among Chinese hypertensive population (31). Although these studies involved METS-IR, the association of METS-IR with CVD mortality has not be investigated. The present study filled this research gap and found that RA patients with increased METS-IR had a significantly higher risk of CVD mortality, and METS-IR>2.48 was associated with a significantly greater risk of CVD mortality than METS-IR ≤ 2.25. METS-IR has been shown to be the most recommended formula to evaluate insulin resistance (32), which is developed with FBG, TG, BMI, and HDL-C. FBG level is a superior predictor of mortality risk and may be applied as a simple predictive and preventative factor (33). The results indicated that higher insulin resistance was associated with a significantly increased risk of CVD mortality among patients with RA. Besides, this study clarified the specific strength of the association between excessive insulin resistance status and increased CVD mortality by setting clear METS-IR thresholds, which may provide a reliable indicator for future evaluation of the risk of CVD mortality in the future. In addition, this also added to current literature on the quantitative relationship between insulin resistance and CVD outcomes.

In patients with RA, the association between METS-IR and the risk of CVD mortality may involve several mechanisms: (1) interaction between inflammation and insulin resistance: RA itself is a systemic inflammatory disease, and chronic inflammation can lead to enhanced insulin resistance (34). Pro-inflammatory cytokines such as TNF-α, IL-6, etc., affect insulin sensitivity in adipose tissue, liver, and muscle (35), thereby increasing METS-IR. Insulin resistance is also closely related to the development and progression of atherosclerosis, thereby escalating the risk of CVDs (36); (2) shared pathophysiological mechanisms: RA patients often present with metabolic syndrome, characterized by hyperglycemia, hypertension, and abnormal lipid profiles, which are all critical risk factors for CVDs (37, 38). METS-IR takes into account both basal metabolic rate and insulin sensitivity (22). A higher METS-IR may imply that patients are concurrently facing greater metabolic abnormalities, thus augmenting the risk of CVD mortality; (3) indirect impact of drug therapy: certain medications used to treat RA, such as glucocorticoids and some disease-modifying anti-rheumatic drugs, may unfavorably affect glucose and lipid metabolism, indirectly causing increased insulin resistance and consequently elevating METS-IR, as well as the risk of cardiovascular events (39); (4) limited physical activity and lifestyle factors: due to joint pain and functional limitations, RA patients often experience restricted physical activity and reduced exercise (40), which not only affects metabolic health but may also exacerbate insulin resistance, manifesting as elevated METS-IR, further contributing to the increased risk of CVDs. Consequently, enhanced attention and management of METS-IR are crucial for improving cardiovascular outcomes in RA patients.

Furthermore, the association between METS-IR and CVD mortality differed across age, gender, BMI, diabetes, and CVD subpopulations. To be noted, among RA patients with BMI less than 30 kg/m², there was an association between METS-IR and the risk of CVD mortality, while in RA patients with BMI ≥ 30 kg/m², no such association was found between METS-IR and CVD mortality. Possible explanations include the following points. In obese RA patients with BMI of 30 kg/m² or higher, they may possess other strong risk factors for CVD, such as dyslipidemia, hypertension, and hyperglycemia (41). These factors could potentially mask the impact of METS-IR on CVD mortality risk. In other words, in an obese population, the significance of METS-IR relative to other risk factors might be relatively smaller. Additionally, RA patients with BMI ≥ 30 kg/m² may require additional medications beyond anti-inflammatory drugs, such as hypoglycemic agents or lipid-lowering drugs (42). These medications could exert different regulatory effects on insulin resistance and CVD risk, thereby influencing the relationship between METS-IR and the risk of CVD mortality risk. For another, BMI is associated with both METS-IR and CVD mortality risk (28). This may make it challenging in statistical analyses to discern the independent contribution of METS-IR to CVD mortality risk. Therefore, while METS-IR might still be related to insulin resistance and metabolic abnormalities in obese RA patients, it may cease to be a decisive predictive marker for the risk of CVD death due to the multiple risk factors and complex physiological and pathological changes brought about by obesity. Further clinical research is required to substantiate and clarify the detailed mechanisms underlying this phenomenon.

The strengths of this study included: the relationship between insulin resistance and CVD death was first explore in RA patients; the long follow-up time ensured the sample size of outcome events (CVD death); the samples were obtained through multi-stage complex sampling from the NHANES database and showed good representativeness. This work reinforces the importance of not only focusing on traditional risk factors when preventing and managing CVDs, but also emphasizing early diagnosis and intervention for insulin resistance. Based on the findings of this study, clinicians should consider incorporating the METS-IR index into routine assessment of CVD mortality risk, particularly for patients whose METS-IR exceeds certain thresholds. Some limitations should be mentioned. On the one hand, information on disease history was obtained through questionnaire surveys, which may be subject to recall bias. On the other hand, due to database constraints, glycemic and lipid indices were obtained from single-time-point measurements. Also, the data were obtained in populations in the United State, and the generalization of the results in other populations should be done with caution. Further investigation is needed to understand the potential influence of changes in METR-IS score on the CVD mortality risk for RA patients.

## Conclusion

Elevated METS-IR levels was associated with CVD mortality Further, the association between METS-IR and CVD mortality varied across age, gender, BMI, diabetes, and CVD subgroups. More studies are warranted to confirm these findings.

## Data Availability

The original contributions presented in the study are included in the article/**Supplementary Material**. Further inquiries can be directed to the corresponding author.
