# Supplementary material for: Association between metabolic score for insulin resistance and cardiovascular disease mortality in patients with rheumatoid arthritis: evidence from the NHANES 1999-2018
Source: Front Endocrinol (Lausanne). 2024 Sep 13;15:1444800. doi: 10.3389/fendo.2024.1444800 (PMC11427302; doi:10.3389/fendo.2024.1444800)
Supplement: Supplementary file 2 [file Table1.docx]

Supplementary Table 1 The number and percentages of missing values

| Variables | n (%) |
| --- | --- |
| Education | 2 (0.16%) |
| Marriage | 9 (0.74%) |
| PIR | 112 (9.2%) |
| Smoke | 2 (0.16%) |
| Drink | 75 (6.16%) |
| Total energy | 46 (3.78%) |
| Physical activity | 509 (41.79%) |
| Duration of arthritis | 12 (0.99%) |
| Osteoporosis | 253 (20.77%) |
| Fracture | 252 (20.69%) |
| CKD | 23 (1.89%) |
| WBC | 6 (0.49%) |
| Uric acid | 7 (0.57%) |

PIR, poverty income ratio; CKD, chronic kidney disease; WBC, white blood cell

Supplementary Table 2 Differences between pre- and post-imputation data

| Variables | Before imputation | After imputation | Statistics | *P* |
| --- | --- | --- | --- | --- |
| Education, n (%) |  |  | χ^2^=1.98 | 0.372 |
| Below high school | 455 (27.20) | 457 (27.22) |  |  |
| High school | 287 (27.18) | 287 (27.17) |  |  |
| College and above | 474 (45.61) | 474 (45.60) |  |  |
| Marital status, n (%) |  |  | χ^2^=1.12 | 0.570 |
| Married | 636 (60.54) | 642 (60.56) |  |  |
| Never married | 88 (6.71) | 89 (6.67) |  |  |
| Others | 485 (32.76) | 487 (32.77) |  |  |
| PIR, n (%) |  |  | χ^2^=0.35 | 0.554 |
| <1.0 | 276 (19.60) | 305 (19.44) |  |  |
| ≥1.0 | 830 (80.40) | 913 (80.56) |  |  |
| Smoking, n (%) |  |  | χ^2^=0.04 | 0.840 |
| No | 528 (38.01) | 529 (38.01) |  |  |
| Yes | 688 (61.99) | 689 (61.99) |  |  |
| Drinking, n (%) |  |  | χ^2^=1.89 | 0.169 |
| No | 418 (33.46) | 455 (33.78) |  |  |
| Yes | 725 (66.54) | 763 (66.22) |  |  |
| Total energy, kcal, Mean (SE) | 2005.01 (41.14) | 2002.83 (40.57) | t=0.59 | 0.553 |
| Duration of arthritis, years, Mean (SE) | 14.06 (0.50) | 14.10 (0.50) | t=-1.58 | 0.115 |
| CKD, n (%) |  |  | χ^2^=1.52 | 0.218 |
| No | 924 (82.66) | 935 (82.45) |  |  |
| Yes | 271 (17.34) | 283 (17.55) |  |  |
| WBC,1000 cells/μL, Mean (SE) | 7.01 (0.09) | 7.01 (0.09) | t=0.76 | 0.448 |
| Uric acid, mg/dL, Mean (SE) | 5.74 (0.06) | 5.74 (0.06) | t=-0.27 | 0.787 |

PIR, poverty income ratio; CKD, chronic kidney disease; WBC, white blood cell; SE, standard error

Supplementary Table 3 Screening of the potential confounding factors associated with CVD mortality in RA patients

| Variables | HR (95%CI) | *P* |
| --- | --- | --- |
| Age | 1.08 (1.05-1.11) | <0.001 |
| Gender |  |  |
| Male | Ref |  |
| Female | 0.86 (0.55-1.35) | 0.503 |
| Race |  |  |
| Non-Hispanic White | Ref |  |
| Non-Hispanic Black | 0.78 (0.45-1.33) | 0.352 |
| Others | 0.81 (0.48-1.37) | 0.430 |
| Education |  |  |
| Below high school | Ref |  |
| High school | 0.81 (0.47-1.42) | 0.468 |
| College and above | 0.50 (0.27-0.92) | 0.026 |
| Marital status |  |  |
| Married | Ref |  |
| Never married | 0.83 (0.30-2.31) | 0.720 |
| Others | 2.31 (1.46-3.66) | <0.001 |
| PIR |  |  |
| <1.0 | Ref |  |
| ≥1.0 | 0.69 (0.44-1.08) | 0.105 |
| Smoke |  |  |
| No | Ref |  |
| Yes | 0.75 (0.47-1.22) | 0.248 |
| Drink |  |  |
| No | Ref |  |
| Yes | 0.83 (0.51-1.35) | 0.454 |
| Total energy | 0.99 (0.99-0.99) | 0.053 |
| Physical activity |  |  |
| <450 MET × minutes/week | Ref |  |
| ≥450 MET × minutes/week | 0.46 (0.22-0.96) | 0.039 |
| Unknown | 1.10 (0.53-2.26) | 0.802 |
| Duration of arthritis | 1.03 (1.01-1.04) | 0.002 |
| Osteoporosis |  |  |
| No | Ref |  |
| Yes | 2.05 (0.99-4.25) | 0.054 |
| Unknown | 0.55 (0.20-1.50) | 0.244 |
| Fracture |  |  |
| No | Ref |  |
| Yes | 1.18 (0.60-2.32) | 0.620 |
| Unknown | 0.51 (0.18-1.42) | 0.194 |
| Diabetes |  |  |
| No | Ref |  |
| Yes | 2.51 (1.43-4.39) | 0.001 |
| Hypertension |  |  |
| No | Ref |  |
| Yes | 3.55 (1.79-7.03) | <0.001 |
| Dyslipidemia |  |  |
| No | Ref |  |
| Yes | 2.13 (1.19-3.83) | 0.012 |
| CVD |  |  |
| No | Ref |  |
| Yes | 2.94 (1.78-4.87) | <0.001 |
| CKD |  |  |
| No | Ref |  |
| Yes | 3.88 (2.36-6.37) | <0.001 |
| Body mass index | 1.02 (1.01-1.04) | 0.096 |
| WBC | 1.07 (1.01-1.13) | 0.013 |
| Uric acid | 1.20 (1.03-1.39) | 0.022 |
| Antirheumatics |  |  |
| No | Ref |  |
| Yes | 0.59 (0.26-1.37) | 0.222 |
| Nonsteroidal anti-inflammatory agents |  |  |
| No | Ref |  |
| Yes | 0.95 (0.49-1.84) | 0.881 |
| Glucocorticoid |  |  |
| No | Ref |  |
| Yes | 1.05 (0.48-2.28) | 0.906 |

RA, rheumatoid arthritis; CVD, cardiovascular disease; METS-IR, Metabolic Score for Insulin Resistance; PIR, poverty income ratio; CKD, chronic kidney disease; BMI, body mass index; WBC, white blood cell; SE, standard error
